# Horizontal gene transfer and diverse functional constrains within a common replication-partitioning system in *Alphaproteobacteria*: the *repABC *operon

**DOI:** 10.1186/1471-2164-10-536

**Published:** 2009-11-18

**Authors:** Santiago Castillo-Ramírez, Jorge F Vázquez-Castellanos, Víctor González, Miguel A Cevallos

**Affiliations:** 1 Programa de Genómica Evolutiva, Centro de Ciencias Genómicas, Universidad Nacional Autónoma de México, Apartado Postal 565-A, CP 62210, Cuernavaca, Morelos, México

## Abstract

**Background:**

The *repABC *plasmid family, which is extensively present within *Alphaproteobacteria*, and some secondary chromosomes of the *Rhizobiales *have the particular feature that all the elements involved in replication and partitioning reside within one transcriptional unit, the *repABC *operon. Given the functional interactions among the elements of the *repABC *operon, and the fact that they all reside in the same operon, a common evolutionary history would be expected if the entire operon had been horizontally transferred. Here, we tested whether there is a common evolutionary history within the *repABC *operon. We further examined different incompatibility groups in terms of their differentiation and degree of adaptation to their host.

**Results:**

We did not find a single evolutionary history within the *repABC *operon. Each protein had a particular phylogeny, horizontal gene transfer events of the individual genes within the operon were detected, and different functional constraints were found within and between the Rep proteins. When different *repABC *operons coexisted in the same genome, they were well differentiated from one another. Finally, we found different levels of adaptation to the host genome within and between *repABC *operons coexisting in the same species.

**Conclusion:**

Horizontal gene transfer with conservation of the *repABC *operon structure provides a highly dynamic operon in which each member of this operon has its own evolutionary dynamics. In addition, it seems that different incompatibility groups present in the same species have different degrees of adaptation to their host genomes, in proportion to the amount of time the incompatibility group has coexisted with the host genome.

## Background

The *repABC *plasmids are a typical genome component of many *Alphaproteobacteria *species. In fact, more than 20 *Alphaproteobacteria *species have at least one *repABC *plasmid (see refs [1,2] for recent reviews), these *repABC *plasmids may be the commonest plasmids in *Alphaproteobacteria *species. In some species these *repABC *plasmids constitute a significant amount of the bacterial genome; such is the case of *Rhizobium leguminosarum *3841, in which *repABC *plasmids account for 35% of the genome [3]. This plasmid family includes several incompatibility groups, meaning that more than one type of *repABC *plasmid can reside in the same bacterial species [1,2]. For instance, *Rhizobium etli *CFN42 has 6 plasmids, all of them *repABC *plasmids [4]. In contrast to other low copy-number plasmids, in which the elements involved in plasmid replication and segregation are located on different loci (each one under its own regulatory circuit), the *repABC *plasmids contain all the elements required for replication and partition within the *repABC *operon. In general, this transcriptional unit comprises three protein-encoding genes (*repA*, *repB*, and *repC*) and a gene encoding a small antisense RNA (ctRNA) [5], which is located within the *repB-repC *intergenic region. The proteins encoded in the *repABC *operon have an intricate relationship, with RepA and RepB interacting both with themselves and with each other. These proteins, in conjunction with the centromere-like sequence, *parS*, function as the plasmid's segregation machinery [1,2,6]. On one hand, RepA is a transcriptional repressor of the operon, while RepB acts as its co-repressor by contacting the operator sequence. The third protein-encoding gene of the operon, *repC*, is essential for plasmid replication; it encodes the initiator protein, RepC, which exerts its function by binding the origin of replication located within its own coding sequence [1,2,6]. Taking these observations into account, it is reasonable to hypothesize that the *repABC *operon is under concerted evolutionary pressures aimed at maintaining its functionality and avoiding incompatibility with other *repABC *operons. Remarkably, this operon is not only the replication system of *repABC *plasmids, but of some secondary chromosomes of some *Rhizobiales *species. For instance, the second chromosomes of *Agrobacterium *vitis S4 and *Agrobacterium tumefaciens *C58 have a *repABC *origin of replication [7].

At the structural level, the various *repABC *operons are only superficially homogeneous; they are highly diverse in DNA sequence, and some possess specific structural elements shared only by few members of the family. These distinctive elements fall into three types: (a) the number and class of regulatory elements involved in operon transcription; (b) the number and position of centromere-like sequences (*parS *sequences); and (c) the presence of peptide-encoding minigenes [1]. Several *Alphaproteobacteria *genomes possess *repAB *genes that are not in close association with the ctRNA or *repC *sequences. However, it has been shown that replication of some *Alphaproteobacteria *plasmids depends only on RepC and a ctRNA, without the involvement of the *repAB *genes. This suggests that fusion of different modules could participate in the generation of new *repABC *plasmids, indicating that the different elements may have experienced different evolutionary histories.

Plasmid stability requires an exquisite balance among all of the interacting molecules involved in plasmid replication and segregation. Perturbation of this balance, for example by the introduction of any replication or segregation element in excess, could lead to plasmid incompatibility. It has been shown that *repABC *plasmids contain at least four elements involved in plasmid incompatibility: the RepA and RepB proteins, the small antisense RNA, and the *parS *sequences [6,8-10]. Phylogenies made with RepA, RepB, and RepC proteins have shown that different replicons residing in the same bacterial strain tend to belong to different clades [11]. Other study found that phylogenetic analyses of *repABC *gene lineages had a lack of evolutionary congruence with the species tree [7]. These observations suggest that divergent evolution followed by episodes of horizontal transfer have played a central role in originating new incompatibility groups. We might therefore expect that incompatibility groups residing in the same genome would be different enough so as to not interfere with each other.

In this study, we analyzed three aspects of *repABC *operons. First, because it is known that *repABC *operon has been horizontally transferred, through phylogenetic analyses, we examined horizontal gene transfer of entire operon versus horizontal transfer of individual genes within this operon. This is a key point, since a previous study has shown that some bacterial operons present horizontal gene transfer events that affect not the entire operons but single genes within the operons [12]. Second, we determined the degree of differentiation among *repABC *operons from different plasmids residing in the same strain (which implies different incompatibility groups). Third, we established the degree of evolutionary adaptedness among different *repABC *operons coexisting in a single species. In principle, because all the elements of the partition and replication systems are contained in the same operon and the encoded proteins interact, these elements might be expected to present almost the same history. Contrary to this, we found significantly different histories for the various elements of the *repABC *operon. Moreover, we detected different selective constraints among the elements composing the operon, and even within individual components. As expected, when different incompatibility groups coexisted in a species, these groups were clearly differentiated from one another. Finally, we found different levels of adaptation to the host genome within and between *repABC *operons coexisting in the same species.

## Results

### The collection of homologous *repABC *operons

To date, at least 81 *repABC *operons have been recognized across the class *Alphaproteobacteria *[1]. Because we wanted to utilize only homologous groups with the same domain structure, we established strict criteria for defining homologous *rep *genes and operons (see Methods). As a result of this, we analyzed only 49 operons herein (see Additional file 1). Twenty-one genomes had at least one *repABC *operon, and most of the operons were located on plasmids. A few genomes, such as those from genera *Brucella *and *Agrobacterium*, had *repABC *operons located on replicons that are considered secondary chromosomes (see Additional file 1). Two *Rhizobium *species, *R. etli *CFN42 and *R. leguminosarum *3841, had the highest number of *repABC *operons, with seven operons each. All plasmids from these species had a single operon, with the exceptions of plasmid p42f from *R. etli *CFN42 and plasmid pRL11 from *R. leguminosarum *3841, which each had two operons per plasmid. We also found six faulty operons that were missing one of the three protein-encoding genes; five out of six were composed of *repA *and *repB *genes, while the remaining one consisted of *repA *and *repC*. In four of six cases, the faulty operons coexisted with complete operons. In many species only one gene was present; by far the most widely distributed gene was *repA*, followed by *repC *(see Additional file 1).

### There is no a single history for the *repABC *operon

**Figure 1:**
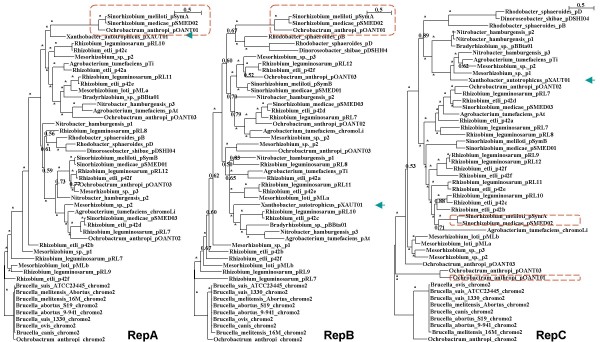
**The individual Bayesian phylogenies**. The Bayesian phylogenies for RepA, RepB, and RepC. The scale bar denotes the estimated number of amino acid substitution per site. The asterisks on the branches represent posterior probability values higher than 0.95, otherwise values are shown. All of the phylogenies were artificially rooted with the homologous gene on chromosome 2 of *Ochrobactrum anthropi*, to facilitate visual comparison between phylogenies.

**Figure 2:**
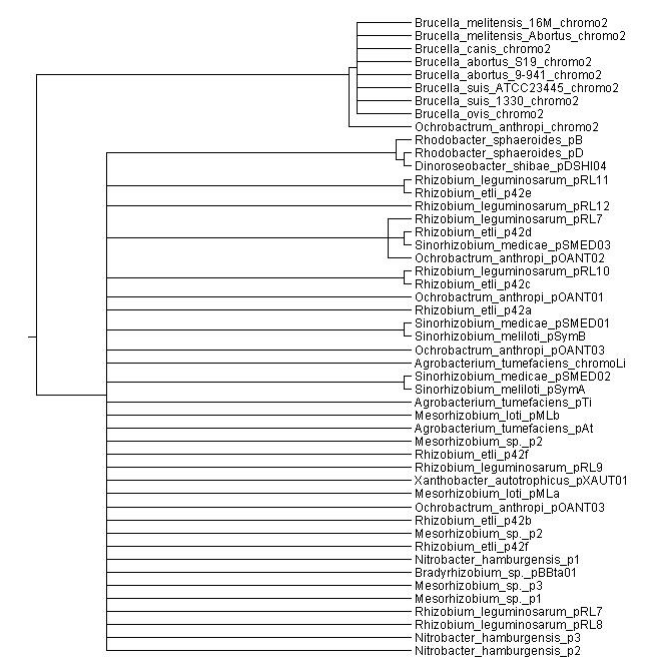
**The strict consensus tree**. A strict consensus tree was constructed using the Bayesian phylogenies of RepA, RepB, and RepC.

Our first goal was to test whether the elements of the *repABC *operon have a common evolutionary history. A single history would be expected if the entire operon had been transferred; on the opposite, if the individual genes were transferred, several histories would be expected. Given that the partition and replication elements functionally interact with each other and compose a single transcriptional unit, we expected to find a single history *a priori*. To test this possibility, we constructed individual Bayesian phylogenies for each protein, and used the phylogenies to construct a strict consensus tree. We obtained phylogenies with strong support, but no two phylogenies gave the same topology (see Figure 1). For example, when we considered the phylogenies for RepA and RepC, only five nodes out of 40 achieved a posterior probability below 0.95 (see Figure 1). There was a large degree of conflict among the individual phylogenies, as demonstrated by the fact that the strict consensus tree had many polytomies and was poorly resolved (Figure 2). Only 25% of the nodes composing each phylogeny were shared among the three phylogenies. Since confidence sets of genes trees have been used to compare competing gene trees [13], we used this method to examine whether the differences among the phylogenies of RepA, RepB, and RepC were more than would be expected by chance (see Methods). The individual alignments of each protein rejected all but its own phylogeny, indicating that the phylogenies of the different proteins were significantly different from each other. Therefore, horizontal gene transfer has affected the individual genes within the operon. Actually each gene has had many unique horizontal gene transfer events since protein alignments rejected all but its own phylogeny. Here we will describe the positions of a couple plasmids in the rep phylogenies to make this clear. First example, the proteins coded by genes of the *repABC *operon located on plasmid pXAUT01 of *Xanthobacter autotrophicus *occupy drastically different positions in all 3 phylogenies (see Figure 1, green arrows). Actually, in each rep phylogeny pXAUT01 clusters with distinct groups, with very good support in every case. In other example, the horizontal gene transfer has affected either 2 of the genes or one gene, as 2 phylogenies agree while the third disagrees; for example, whereas the plasmids pSymA and pSMED02 cluster with pOANT01, in RepA and RepB phylogenies, plasmid pOANT01 does not cluster with the other 2 in RepC phylogeny (see Figure 1, red squares). The horizontal gene transfer events that have affected the *rep *genes are very particular, as they did not disrupt the operon structure. Gene displacement *in situ *is the most probable process behind this observation given that the operon is conserved in all the cases. As expected, the phylogenies for RepA and RepB, whose genes are next to each other, were more alike to one another than to RepC, as the Robison-Fould distance (a metric used to compare phylogenies, in which increasing distance indicates increasing disparity between phylogenies) between the phylogenies of RepA and RepB was smaller than that between RepC and either RepA or RepB (see Additional file 2). Since the evolutionary distance within the RepA, RepB, and RepC phylogenies is not that vast (see Figure 1), we checked if *in situ *gene displacement occurred by means of homologous recombination. To see if this might be the case here, we performed recombination analyses on the DNA alignments. In all three genes we found evidence of recombination, pairwise identity plots of the localized recombination events are presented in Additional file 3. We identified one event for *repA*, two for *repB*, and up to four for *repC *(see Additional files 3 and 4). The above results suggest that *in situ *gene displacement within the operon, through homologous recombination, has affected the *repABC *operon.

### Different levels of functional restriction within and between Rep proteins

The most common method for modeling the variation of evolutionary rates among sites is the gamma distribution. Its shape parameter, α, determines the extent of rate variation among sites; a small α represents extreme rate variation, while a large α value represent a minor variation in rate [14]. Given that the main reason for the heterogeneity of evolutionary rates among sites seems to be differences in their selective constraints (due to the functional and/or structural requirements of the gene), we herein used the shape parameter α as a proxy for the functional restriction of each studied protein. In addition, we used the total length of each phylogeny as a proxy to examine the level of protein conservation. Among the three studied proteins, RepA showed the lowest total phylogenetic length and the highest among-site rate variation (reflected through the smallest shape parameter α), indicating that RepA was the most conserved protein, and that it experienced the highest level of functional restriction. The confidence intervals of the total length of the RepA phylogenies did not overlap with those of the two other phylogenies (see Table 1). Interestingly, the among-site rate variation was not significantly different between RepA and RepC, but the among-site rate variations of these two proteins were significantly different from that of RepB (see Table 1, shape parameter α column). Therefore, although RepA was the most conserved protein, RepA and RepC had similar levels of functional restriction.

**Table 1:** Estimates of the best amino acid models for the individual Bayesian phylogenies

*Protein*	*TL*	*Shape parameter α*	*P. Invariant sites*	*Model*
**RepA**	11.987 (11.128 12.906)	0.933 (0.776 1.102)	0.065 (0.022 0.107)	WAG (PP 1)

**RepB**	19.952 (18.617 21.323)	1.721 (1.487 1.983)	0.0698 (0.041 0.103)	WAG (PP 1)

**RepC**	17.678 (16.49 18.922)	1.122 (0.993 1.265)	0.068 (0.040 0.098)	JTT (PP 1)

The amino acid matrix with the highest posterior probability, the estimated proportion of invariable sites, and the estimated gamma shape parameter for each Rep protein.

Abbreviations. TL: total length of the phylogeny, PP: posterior probability. The values in parentheses is the 95% Cred. Interval.

**Figure 3:**
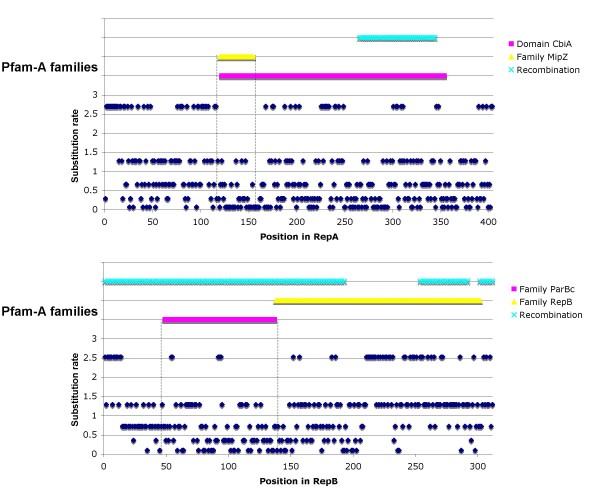
**Functional restrictions within the RepA and RepB proteins**. Substitution rate variation among sites in the RepA and RepB proteins. For each protein, all sites were assigned to one of five gamma categories. The Pfam-A domains are shown for each protein, as well as the zones affected by recombination events.

To assess functional restriction inside the proteins, we next identified domains using Pfam [15], and assigned substitutions rates for individual sites for each protein using a discrete-gamma distribution (see Methods). We found that different domains had different substitution rates. For instance, in RepA protein, the ATPase domain almost did not have positions with highest substitution rates (see Figure 3, dotted lines, family MipZ), whereas the nucleotide-binding domain did have positions with the highest substitution rates (see Figure 3, domain CbiA). Similarly, most of the sites in the ParB-like nuclease domain of RepB (see Figure 3, dotted lines, family ParBc) had substitution rates that were smaller than those of the plasmid partition family domain (see Figure 3, family RepB). Only one domain was identified for RepC, but the substitution rates of its sites varied (Additional file 5). Notably, whereas RepA (the most conserved protein) was affected by a recombination event within its more variable domain (see Figure 3, Recombination, upper panel), RepB seems to have been affected by recombination throughout its sequence (see Figure 3, Recombination, lower panel). Thus, we detected different levels of functional restriction not only between the studied proteins, but also within them.

### Well differentiated incompatibility groups

We used *Rhizobium etli *CFN42 and *Rhizobium leguminosarum *3841 to compare and contrast incompatibility groups, because these strains each harbored six *repABC *compatible plasmids (i.e., six incompatibility groups). We made four DNA alignments, one for each *rep *gene and one for the intergenic region between the *repB *and *repC *genes, which encodes a small antisense RNA gene that acts as a strong incompatibility factor. To evaluate the degree of distinction among the *rep *genes and intergenic region of the different incompatibility groups, we determined maximum likelihood matrices and then calculated the average distance over all possible pairs of sequences (see Methods). The genes and intergenic region could be clearly differentiated across the different plasmids (see Table 2). In agreement with our protein phylogenies, the *repA *and *repC *genes presented shorter average distances and higher proportions of invariant sites compared to *repB*. Notably, the intergenic region comprised the shortest distance, but did not have any invariant position (see Table 2). Moreover, this locus had the highest among-site rate variation, as reflected in the smallest shape parameter α (see Table 2). This suggests that the intergenic region is under higher functional restriction compared to the *rep *genes; this finding is compatible with the presence of the small antisense RNA-encoding sequence in the intergenic region. Neither the intergenic region nor the *rep *genes showed any evidence of recombination. These results suggest that there was a high degree of differentiation among the examined incompatibility groups.

**Table 2:** Average between-locus distance for the different loci

*Locus*	*Average distance+*	*P. Invariant sites*	*Shape parameter α*
***repA***	0.72530	0.194	1.176

***repB***	1.13479	0.089	1.661

**Intergenic region**	0.45827	0.0	0.47

***repC***	0.59197	0.186	1.108

The average distance over all possible sequence pairs for each locus, along with the specifications made by jModelTest regarding the substitution model.

+Average distance over all possible pairs of sequences.

All the loci but the "Intergenic region" selected the GTR model with correction for across site rate variation and invariant sites (GTR+I+G). The "Intergenic region" selected *TPM2 with correction for across site rate variation (TPM2+G).

*This model implies AC = AT; CG = GT; AG = CT;

### Codon Adaptation Index as a measure of evolutionary adaptedness

**Figure 4:**
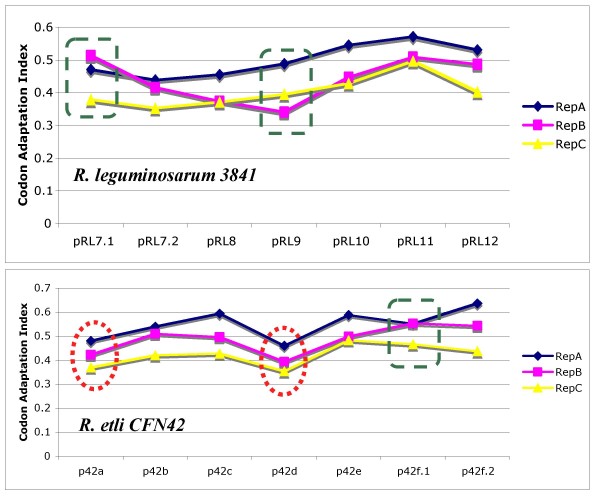
**Codon Adaptation Index**. CAI values are shown for each of the genes comprising the *repABC *operons found in *R. etli *CFN42 and *R. leguminosarum *3841. Red circles indicate the putatively newest plasmids in *R. etli *CFN42. Green squares show the inconsistencies found herein.

The Codon Adaptation Index (CAI) is a simple measure of synonymous codon usage bias. This index uses a reference set of highly expressed genes to assess the relative merits of every codon, and then determines a score for the gene or genes in question based on the use frequency of all codons in that gene [16]. The CAI can be used to evaluate the extent to which selection has been effective in molding the pattern of codon usage [16], and compare the codon usage of foreign genes versus that of highly expressed native genes. Here, we used the CAI to assess the adaptation of the *repA*, *repB*, and *repC *genes to their host genomes. We first calculated the relative synonymous codon usage values of highly expressed native genes (those encoding ribosomal proteins from each species), and then used CAI to compare the codon usage of the *repA*, *repB*, and *repC *genes to those of the reference genes (see Methods). CAI values can range from 0 (reflecting equal use of synonymous codons) to 1 (reflecting the strongest bias, codon usage is equal to that in the reference ribosomal protein-encoding genes). We found a clear trend in the CAI values within and between the studied *repABC *operons. In general, *repA *genes had the highest CAI values, followed by *repB *genes (see Figure 4). The *repABC *operons located on different plasmids had different CAI values, with those located on plasmids appearing to be the newest (e.g., p42a and p42d in *R. etli *CFN42, see Discussion) having the smallest CAI values (see Figure 4, red circles). Notably, in plasmids harboring two *repABC *operons, one always failed to meet the abovementioned pattern of CAI stratification. For example, plasmid pRL7 from *R. leguminosarum *3841 contained the pRL7.1 and pRL7.2 operons, and the former had a higher CAI value for *repB *than *repA *(see Figure 4, green squares). Given that the degree of codon bias in unicellular organisms correlates with the level of gene expression, our results suggest that *repA *is more highly expressed than the other two genes, and *repB *is expressed at a higher level than *repC*. Furthermore, it seems that the different operons have different levels of expression.

## Discussion

The *repABC *operon is not only important because it is the replication-partition system of *repABC *plasmids, a common component of *Alphaproteobacteria *species, but because it is also the replication-partition system of some secondary chromosomes in *Alphaproteobacteria *species. Our present analyses functioned at two levels: within the *repABC *operon and between *repABC *operons in those cases where several *repABC *operons coexisted in the same genome. We did not find a single history within the *repABC *operon; clearly, each protein had its own phylogeny. This is somewhat surprising, since *repA*, *repB*, and *repC *form an operon, and it would seem that they should have similar histories if the entire operon had been horizontally transferred. Instead, even RepA and RepB, which compose the partition system and physically interact, had different phylogenies. This contrast with a recent work in which relaxase sequences were used as tools for classification of conjugative systems. In that study it was found that relaxases and the IV coupling proteins (T4CP), which map next to each other and belong to a minimal gene set that allows plasmid to be conjugally transmitted, evolve congruently for long periods of time [17]. Thus, it seems that compared with some elements of the transfer machinery the *repABC *replication-partition system is highly diverse.

Quite notably, every single gene of this operon presented evidence of horizontal gene transfer. *In situ *gene displacement is a likely process behind this, since the structure of the *repABC *operon is completely conserved. We think *in situ *gene displacement could have occurred through homologous recombination, as we found homologous recombination events across the 3 *rep *genes. Although *in situ *gene displacement appears unlikely, there is evidence that shows that this process is not that scarce. Omelchenko *et al *found that within the bacterial operons they had analyzed *in situ *gene displacement was a frequent event [12]. A striking difference between *in situ *gene displacement and other types of horizontal gene transfer events is that the former leaves intact the operon structure, so that, the operon is completely functional.

The proteins differed not only at the topological level, but also at the level of functional restriction. RepA and RepC, which belong to different systems, were under similar levels of functional restriction, suggesting that key elements of the partitioning and replication systems are under similar functional restrictions. In contrast, RepB had a very different level of functional restriction. We also found different levels of functional restrictions within proteins. For example, the ATPase domain of RepA (Figure 3, family MipZ), which forms a complex with the chromosome partitioning protein and is indispensable for partitioning, presented the lowest substitution rates. As well the only recombination event presented in *repA *did not affect the ATPase domain but a relatively unconserved part of the gene. Therefore, it seems that the different proteins, and even the different parts of the proteins themselves, are under different functional and/or structural constraints. Of the three genes studied, *repA *was the most conserved and might have the highest expression level. This is not unexpected, as RepA is known to have several functions, and its expression is required in both the presence and absence of partition, suggesting the need for high-level translation in order to maintain sufficient RepA levels. In contrast, *repC*, which is a replication initiator protein, had the lowest CAI values, perhaps due to the higher levels of homologous recombination in this gene (see below). Horizontal gene transfer could be very important in allowing the variability of this operon. Indeed, if horizontal gene transfer had not affected the genes within the operon, these genes would have to have a single evolutionary history. Instead, we found that the reverse was true. The proteins encoded in those genes not only presented different phylogenies, they also had different functional restrictions, even within the proteins themselves, and the CAI values differed among the genes. Given the presence of differences at several levels, it is very logical to think that horizontal gene transfer has unconnected the various portions of the operon, allowing each part to have a particular evolutionary history. In this way, genes with very different functional restrictions could be located next to each other, as seen for *repB *and *repA*.

The existence of multiple *repABC *operons located on different replicons in the same genome implies the presence of different incompatibility groups. We herein showed that when multiple *repABC *operons coexisted in the same genome, they were well differentiated from one another. We did not find evidence of homologous recombination in these cases; this is not unexpected, since homologous recombination would homogenize the sequences, meaning that the different groups would no longer be compatible with each other. The intergenic region, which encodes a small antisense RNA (a very important determinant for incompatibility), was highly conserved and found to be under high functional restriction, yet it did not have any invariant sites. Although this sequence has changed only minimally due to functional restrictions, it has still accumulated sufficient changes to allow the coexistence of the different incompatibility groups. In agreement with our within-operon analysis, *repA *and *repC *were highly conserved, with *repC *being the most highly conserved between operons (it had the smallest average distance). As mentioned above, *repC *also had the most homologous recombination events. This suggests that homologous recombination might be reducing the divergence of *repC*, potentially also explaining the low CAI values for this gene (homologous recombination would be erasing any improvement in the CAI values). In a report on the genome sequence of *R. leguminosarum*, Young and coworkers suggested that a recent recombination event had taken place, and divergence of RepC was not critical for plasmid compatibility [3]. Here, one of the recombination events detected in *repC *involved the sequence from pRL8, which is a plasmid of *R. leguminosarum *3841.

Different *repABC *operons had distinct levels of adaptation to their host genome, with no two *repABC *operons presenting the same CAI values. We think that amelioration might be playing a role in the adaptation of *repABC *operons to their hosts. Plasmids p42a and p42d were suggested to be newly acquired plasmids based on their lower GC values, poor conservation, and poor functional connectivity with the rest of the genome [4]. These two plasmids had the worst CAI values, implying that they are not well adapted to their host's genome. In contrast, the operon from p42f, which appeared to be the oldest plasmid harbored within *R. etli *CFN42, had the highest CAI values, suggesting that this operon is highly adapted. These findings indicate that the longer a *repABC *operon coexists with its host genome, the more adapted the operon becomes. This may result in more effective replication and partitioning processes. As well plasmids, which had the most adapted operons, presented essential genes as well; for instance plasmids pRL11, pRL12, and pRL10, which all have essential genes [3], had the operons with higher CAI values than the rest of plasmid of *R. leguminosarum *3841.

## Conclusion

In summary, we herein report finding different histories and functional constraints within the *repABC *operon. In addition, when multiple *repABC *operons were present in the same genome, they had different levels of adaptedness to the host genome, and this seems to be related to the length of time each operon had been associated with the host genome. Finally, horizontal gene transfer with conservation of the operon structure provides a highly dynamic operon in which each member could have its own evolutionary dynamics.

## Methods

### Detection of homologous genes and operons

We first identified the homologous of the RepA, RepB, and RepC proteins across the known *Alphaproteobacteria *genomes (see Additional file 6). The RepA, RepB, and RepC proteins from symbiotic plasmids of *R. etli *CFN42 and *S. meliloti *1021 were used as seeds, and were queried against the proteomes encoded by the other genomes (Additional file 6), using BLAST [18] with an E-value cutoff of 1.0e-12. We retained all cases where a seed protein had a hit in any other proteome and the proteins aligned along at least 70% of their lengths. We then selected for DNA sequences wherein *repA *was next to *repB*, and *repB *was next to *repC *(by definition, the only gene between *repA *and *repC *was *repB*), this was taken as a complete operon. The homologous protein groups contained only proteins whose genes formed complete operons. For each homologous protein group, we constructed an alignment with MUSCLE [19], and used this alignment to infer a phylogeny (see below). To generate the DNA alignments of *repA*, *repB*, and *repC*, we used their protein alignments as references, and performed nucleotide alignment using the "tranalign" program from The European Molecular Biology Open Software Suite (EMBOSS) [20]. The recombination analysis was carried out on these DNA alignments.

Other sets of DNA alignments were created for each of the operons contained in *R. etli *CFN42 and *R. leguminosarum *3841. The intergenic region between *repB *and *repC *was also considered. We then used jModelTest [21] to carry out statistical selection of the best-fit models of nucleotide substitution for every DNA alignment. Finally, maximum likelihood distance matrices were inferred using the model specifications from jModelTest; this was done with PUZZLE [22].

### Phylogenetic Analysis

Phylogenies were created using MrBayes v3.1.2 [23], allowing the MCMC sampler to explore all of the fixed-rated amino acid models included in MrBayes. The number of rate categories for gamma distributions was set to four, with a proportion of sites allowed to be invariable. We performed two runs with four chains each, for 5,000,000 generations. Trees were sampled every 1000 generations, 20% of all generations were removed as burn-in, and a consensus tree was taken. We also estimated the best amino acid models, including the amino acid matrices with the highest posterior probability, estimates of the proportion of invariable sites, and estimates of the gamma shape parameter.

A strict consensus tree was created from all three Bayesian phylogenies, using CONSENSE [24].

We established the similarities of the phylogenies using the Robinson and Fould distance (RFd), as calculated with TREEDIST [24].

We used confidence sets to assess whether the differences in topology between the individual Bayesian phylogenies exceeded those expected to occur by chance. We used expected likelihood weighting [13], which provides a simple and intuitive method for making multiple comparisons of models and constructing the corresponding confidence sets. This test has the benefit of being less conservative than the Shimodaira-Hasegawa test [13]. The topologies tested included those from the RepA, RepB, and RepC phylogenies. PUZZLE [22] was used to carry out this test for each protein alignment.

### Recombination analysis

Although methods that use the substitution patterns or incompatibilities among sites seem be the most powerful strategy for identifying the presence of recombination events, no single method seems to perform optimally under all different scenarios [25]. Thus, the best strategy is often to use a combination of methods. Here, we used the RDP3 program [26], which implements a number of methods for identifying recombination events, including GENECONV [27], RDP [26], MaxChi [28], Chimera [28], SisCan [29], and Bootscanning [30]. We identified a recombination event as valid when at least three of the six methods indicated positive findings.

### Functional regions and among-site rate variation in Rep proteins

We identified the various protein domains by applying the Pfam-A component of Pfam [15]. For this analysis, the RepA, RepB, and RepC proteins of symbiotic plasmid p42d from *R. etli *CFN42 were queried against Pfam-A. For every position of each protein alignment, a substitution rate was assigned using a discrete-gamma distribution. The discrete-gamma distribution used five rate classes and was implemented through PUZZLE.

### Codon Adaptation Index as measure of evolutionary adaptedness

This analysis was done only for the *repA*, *repB*, and *repC *genes located on operons found within species *R. etli *CFN42 and *R. leguminosarum *3841. We used the utility "cusp" from EMBOSS to calculate a codon usage table for the genes encoding the ribosomal proteins in each species. Using these tables as a reference, we applied the "cai" program of the EMBOSS suite to calculate Codon Adaptation Indices for the *repA*, *repB*, and *repC *genes.

## Authors' contributions

SC-R conceived and designed the experiments. SC-R and JFV-C performed the experiments. SC-R analyzed the data. SC-R and MAC discussed the results. MAC and SC-R wrote the manuscript. JFV-C and VG checked the manuscript. VG contributed materials. All authors read and approved the final manuscript.

## Supplementary Material

Additional file 1 **Homologous genes and *repABC *operons**. Homologous genes of *repA, repB*, and *repC*, as well as complete and faulty *repABC *operons found across the studied *Alphaproteobacteria *genomes. For each gene it was registered whether it was located on a chromosome (C) or a plasmid (P). Click here for file

Additional file 2 **Robison-Fould distances between Rep phylogenies**. In order to determine the similarity among the Rep phylogenies, Robison-Fould distances between Rep phylogenies were established. Click here for file

Additional file 3 **Recombination events identified for *repA*, *repB*, and *repC***. Pairwise identity plots of the localized recombination events, showing major and minor parent sequences as well as the daughter sequence. Abbreviations are given in Additional file 4. Click here for file

Additional file 4 **Abbreviations of additional file **3. Abbreviations used in the pairwise identity plots in additional file 3. Click here for file

Additional file 5 **Functional restrictions within RepC**. Substitution rate variation among sites in RepC. All sites were assigned to one of five gamma categories. Pfam-A domains are shown, as well as the zone affected by recombination events. Click here for file

Additional file 6 **Genomes used**. *Alphaproteobacteria *genomes used to search for *repABC *operons. Click here for file
