# Supplementary material for: Horizontal gene transfer and diverse functional constrains within a common replication-partitioning system in Alphaproteobacteria: the repABC operon
Source: BMC Genomics. 2009 Nov 18;10:536. doi: 10.1186/1471-2164-10-536 (PMC2783167; doi:10.1186/1471-2164-10-536)
Supplement: Additional file 1 — Homologous genes and repABC operons. Homologous genes of repA, repB, and repC, as well as complete and faulty repABC operons found across the studied Alphaproteobacteria genomes. For each gene it was registered whether it was located on a chromosome (C) or a plasmid (P). [file 1471-2164-10-536-S1.DOC]

**Additional file 1**

**Genomes in which were found homologous genes of RepA, RepB, and RepC,**

**as well as complete and faulty RepABC operons.**

| Genomes | repABC totales | Genes |  | RepABC operons | Faulty operons |
| --- | --- | --- | --- | --- | --- |
|  | repA | repB | repC |  |  |
| Jannaschia sp. CCS1 | C(0)P(1) |  |  |  |  |
| Novosphingobium aromaticivorans DSM 12444 | C(0)P(2) |  |  |  |  |
| Paracoccus denitrificans PD1222 | C(2)P(1) |  |  |  |  |
| Rhodobacter sphaeroides ATCC 17025 | C(0)P(3) |  |  |  |  |
| Rhodobacter sphaeroides ATCC 17029 | C(1)P(1) |  |  |  |  |
| Silicibacter pomeroyi DSS-3 | C(0)P(1) |  |  |  |  |
| Silicibacter sp. TM1040 | C(0)P(2) |  |  |  |  |
| Sphingomonas wittichii RW1 | C(0)P(1) |  |  |  |  |
| Gluconobacter oxydans 621H | C(0)P(1) | C(0)P(1) |  |  | repAB(1) |
| Methylobacterium sp. 4-46 | C(2)P(0) | C(2)P(0) |  |  | repAB(2) |
| Acidiphilium cryptum JF-5 | C(0)P(1) |  | C(0)P(4) |  |  |
| Agrobacterium tumefaciens str. C58 | C(1)P(2) | C(1)P(2) | C(1)P(2) | 3 |  |
| Bartonella tribocorum CIP 105476 |  |  | C(0)P(1) |  |  |
| Beijerinckia indica subsp. indica ATCC 9039 | C(0)P(1) |  | C(0)P(1) |  |  |
| Bradyrhizobium sp. BTAi1 | C(0)P(1) | C(0)P(1) | C(0)P(1) | 1 |  |
| Brucella abortus biovar 1 str. 9-941 | C(1)P(0) | C(1)P(0) | C(1)P(0) | 1 |  |
| Brucella abortus S19 | C(1)P(0) | C(1)P(0) | C(1)P(0) | 1 |  |
| Brucella canis ATCC 23365 | C(1)P(0) | C(1)P(0) | C(1)P(0) | 1 |  |
| Brucella melitensis 16M | C(1)P(0) | C(1)P(0) | C(1)P(0) | 1 |  |
| Brucella melitensis biovar Abortus 2308 | C(1)P(0) | C(1)P(0) | C(1)P(0) | 1 |  |
| Brucella ovis ATCC 25840 | C(1)P(0) | C(1)P(0) | C(1)P(0) | 1 |  |
| Brucella suis 1330 | C(1)P(0) | C(1)P(0) | C(1)P(0) | 1 |  |
| Brucella suis ATCC 23445 | C(1)P(0) | C(1)P(0) | C(1)P(0) | 1 |  |
| Caulobacter sp. K31 | C(0)P(1) |  | C(0)P(1) |  |  |
| Dinoroseobacter shibae DFL 12 | C(0)P(5) | C(0)P(1) | C(0)P(3) | 1 |  |
| Mesorhizobium sp. BNC1 | C(0)P(4) | C(0)P(4) | C(0)P(5) | 4 |  |
| Mesorhizobium loti MAFF303099 | C(0)P(2) | C(0)P(2) | C(0)P(2) | 2 |  |
| Methylobacterium radiotolerans JCM 2831 | C(0)P(1) | C(0)P(1) | C(0)P(1) |  |  |
| Nitrobacter hamburgensis X14 | C(0)P(3) | C(0)P(3) | C(0)P(3) | 3 |  |
| Ochrobactrum anthropi ATCC 49188 | C(1)P(4) | C(1)P(4) | C(1)P(5) | 5 |  |
| Rhizobium etli CFN 42 | C(0)P(8) | C(0)P(7) | C(0)P(8) | 7 |  |
| Rhizobium leguminosarum bv. viciae 3841 | C(0)P(9) | C(0)P(7) | C(0)P(8) | 7 | repAC(1) |
| Rhodobacter sphaeroides 2.4.1 | C(1)P(4) | C(0)P(2) | C(0)P(3) | 2 |  |
| Roseobacter denitrificans | C(0)P(2) |  | C(0)P(1) |  |  |
| Sinorhizobium medicae WSM419 | C(0)P(4) | C(0)P(4) | C(0)P(3) | 3 | repAB(1) |
| Sinorhizobium meliloti 1021 | C(0)P(3) | C(0)P(3) | C(0)P(2) | 2 | repAB(1) |
| Xanthobacter autotrophicus Py2 | C(0)P(1) | C(0)P(1) | C(0)P(1) | 1 |  |
|  |  |  |  |  |  |
| TOTAL | 85 | 55 | 65 | 49 | 6 |

**C: located on chromosome**

**P: located on plasmid**
