# Supplementary material for: Horizontal gene transfer and diverse functional constrains within a common replication-partitioning system in Alphaproteobacteria: the repABC operon
Source: BMC Genomics. 2009 Nov 18;10:536. doi: 10.1186/1471-2164-10-536 (PMC2783167; doi:10.1186/1471-2164-10-536)
Supplement: Additional file 2 — Robison-Fould distances between Rep phylogenies. In order to determine the similarity among the Rep phylogenies, Robison-Fould distances between Rep phylogenies were established. [file 1471-2164-10-536-S2.DOC]

Tree distance program, version 3.61

Robinson and Fould distances between Rep genes:

RepA RepB RepC

\---------------------------------

RepA | 0 34 46

RepB | 34 0 44

RepC | 46 44 0
