# Supplementary material for: Horizontal gene transfer and diverse functional constrains within a common replication-partitioning system in Alphaproteobacteria: the repABC operon
Source: BMC Genomics. 2009 Nov 18;10:536. doi: 10.1186/1471-2164-10-536 (PMC2783167; doi:10.1186/1471-2164-10-536)
Supplement: Additional file 3 — Recombination events identified for repA, repB, and repC. Pairwise identity plots of the localized recombination events, showing major and minor parent sequences as well as the daughter sequence. Abbreviations are given in Additional file 4. [file 1471-2164-10-536-S3.PPT]

## Slide 1
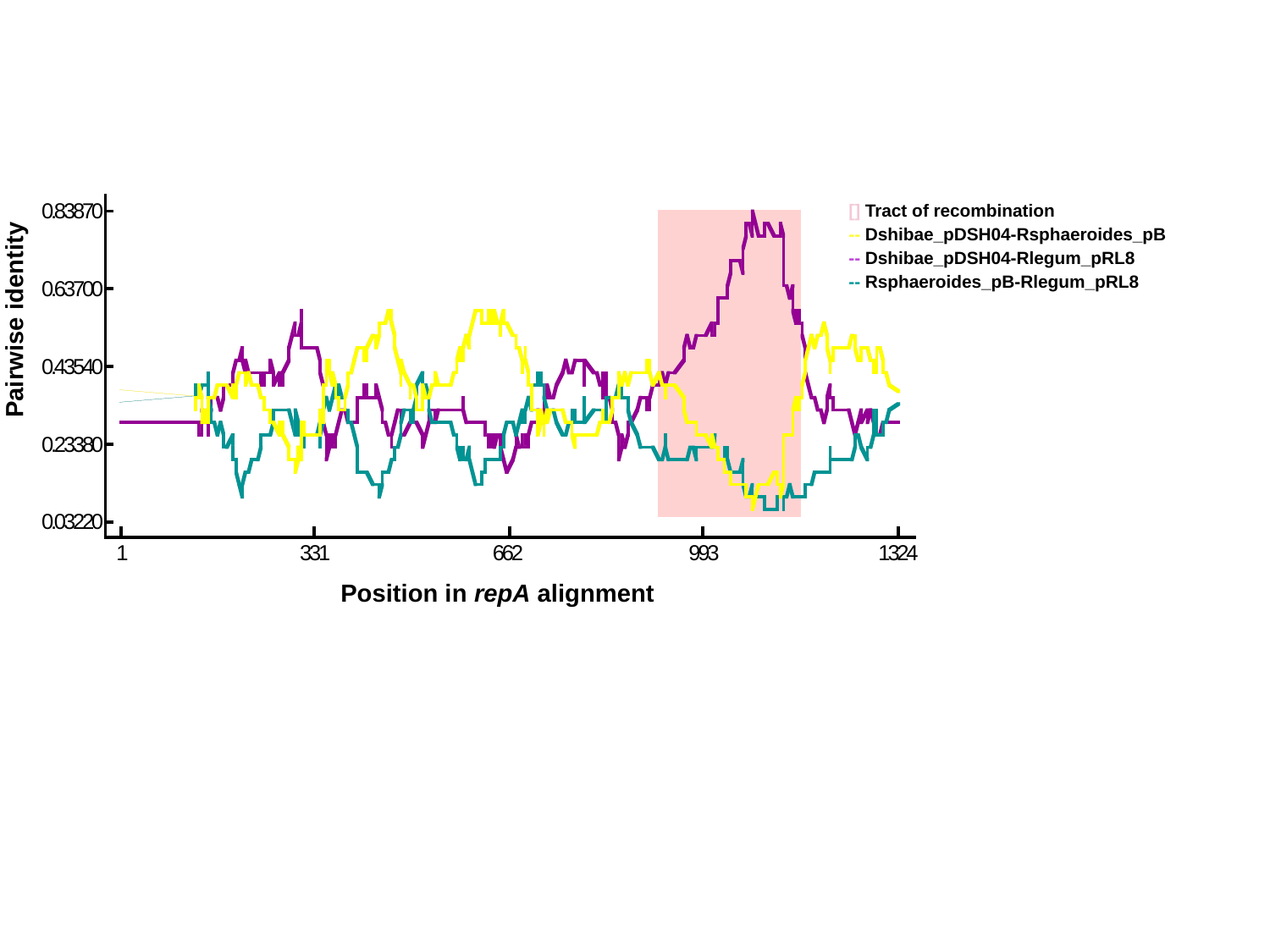

[] Tract of recombination
-- Dshibae_pDSH04-Rsphaeroides_pB
-- Dshibae_pDSH04-Rlegum_pRL8
-- Rsphaeroides_pB-Rlegum_pRL8
Pairwise identity
Position in repA alignment

## Slide 2
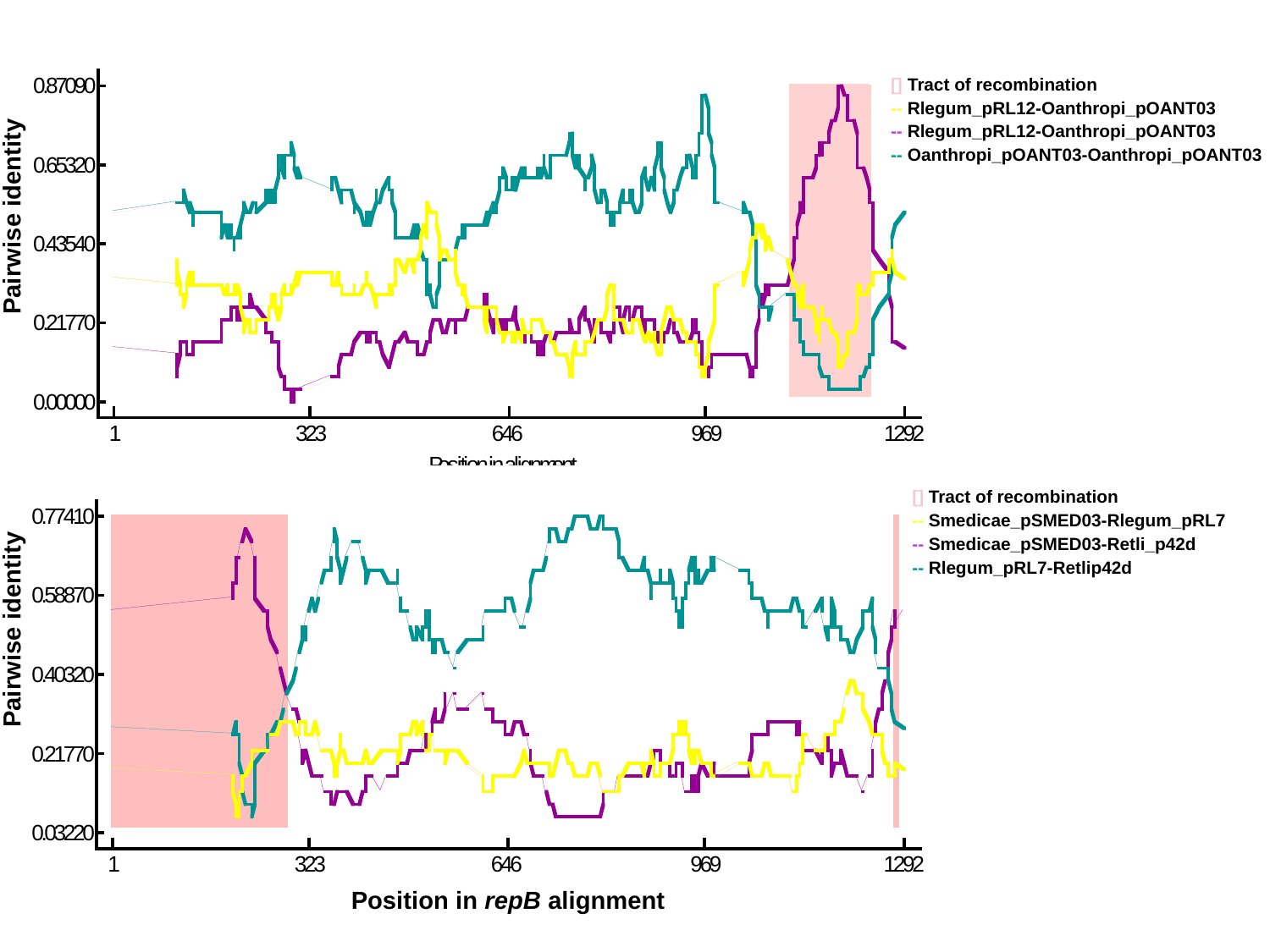

[] Tract of recombination
-- Rlegum_pRL12-Oanthropi_pOANT03
-- Rlegum_pRL12-Oanthropi_pOANT03
-- Oanthropi_pOANT03-Oanthropi_pOANT03
Pairwise identity
[] Tract of recombination
-- Smedicae_pSMED03-Rlegum_pRL7
-- Smedicae_pSMED03-Retli_p42d
-- Rlegum_pRL7-Retlip42d
Pairwise identity
Position in repB alignment

## Slide 3
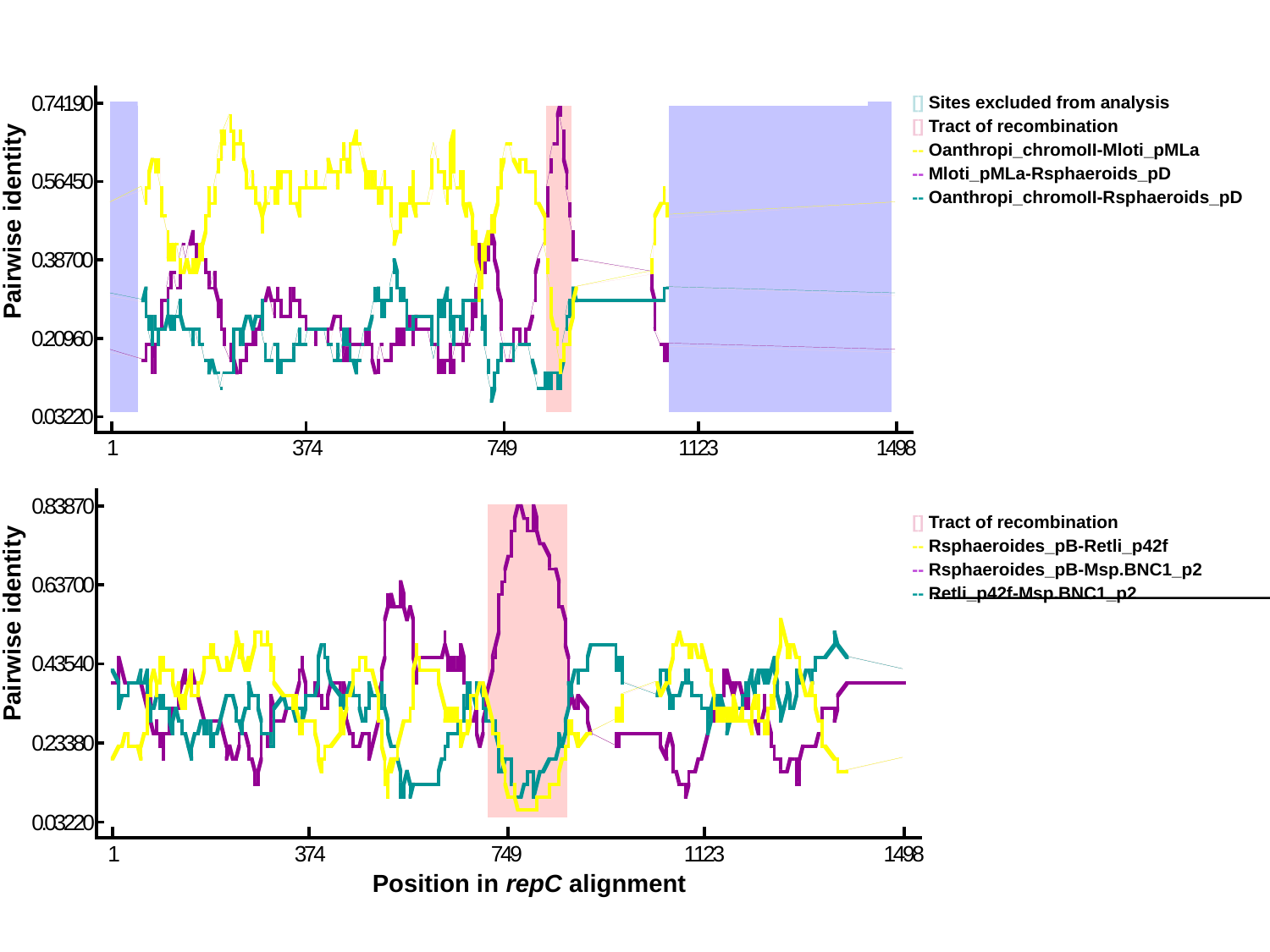

[] Sites excluded from analysis
[] Tract of recombination
-- Oanthropi_chromoII-Mloti_pMLa
-- Mloti_pMLa-Rsphaeroids_pD
-- Oanthropi_chromoII-Rsphaeroids_pD
[] Sites excluded from analysis
[] Tract of recombination
-- Rsphaeroides_pB-Retli_p42f
-- Rsphaeroides_pB-Msp.BNC1_p2
-- Retli_p42f-Msp.BNC1_p2
Pairwise identity
[] Tract of recombination
-- Rsphaeroides_pB-Retli_p42f
-- Rsphaeroides_pB-Msp.BNC1_p2
-- Retli_p42f-Msp.BNC1_p2
Pairwise identity
Position in repC alignment

## Slide 4
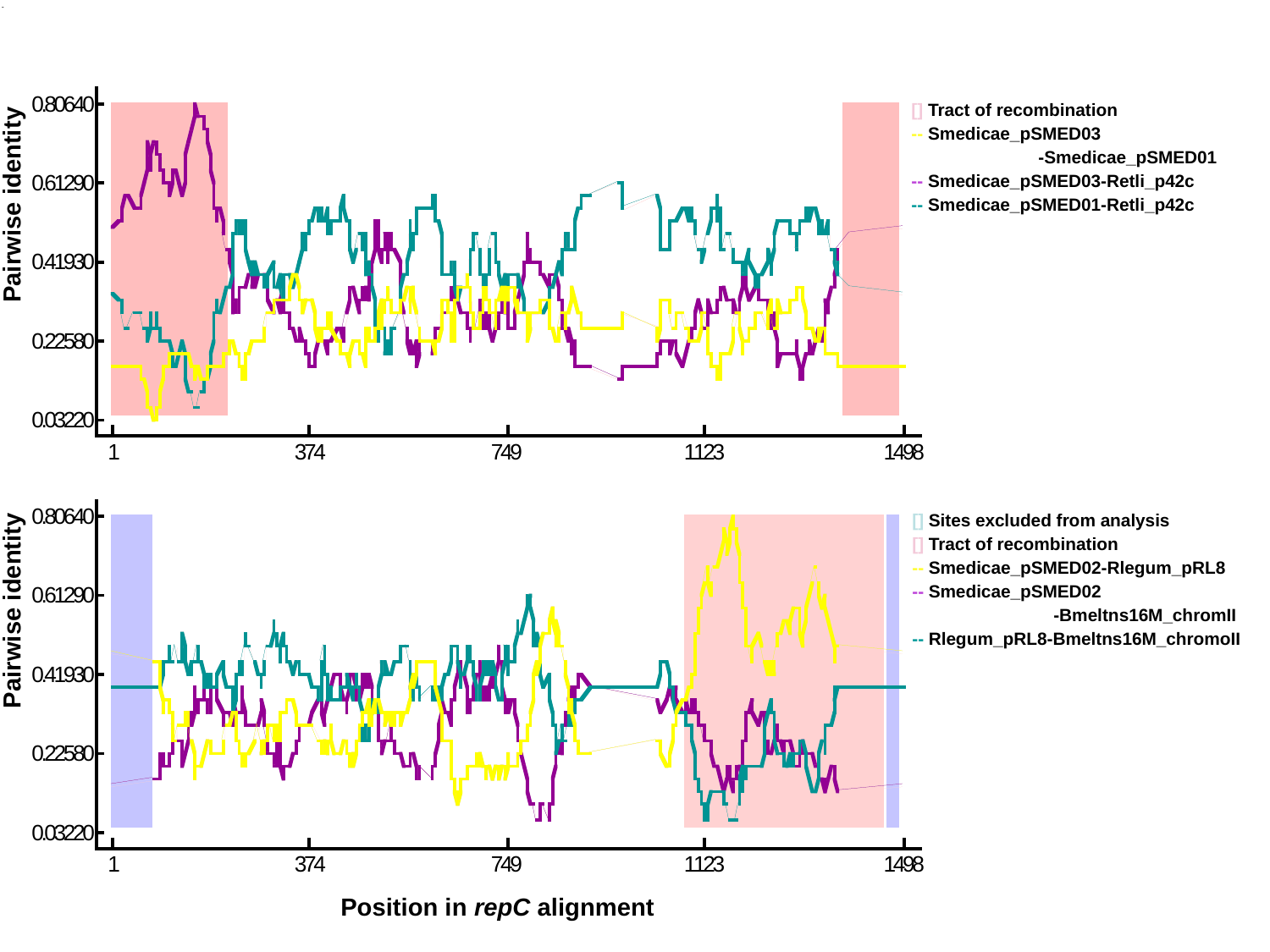

[] Tract of recombination
-- Smedicae_pSMED03
	-Smedicae_pSMED01
-- Smedicae_pSMED03-Retli_p42c
-- Smedicae_pSMED01-Retli_p42c
Pairwise identity
[] Sites excluded from analysis
[] Tract of recombination
-- Smedicae_pSMED02-Rlegum_pRL8
-- Smedicae_pSMED02
	 -Bmeltns16M_chromII
-- Rlegum_pRL8-Bmeltns16M_chromoII
Pairwise identity
Position in repC alignment
