# Supplementary material for: Horizontal gene transfer and diverse functional constrains within a common replication-partitioning system in Alphaproteobacteria: the repABC operon
Source: BMC Genomics. 2009 Nov 18;10:536. doi: 10.1186/1471-2164-10-536 (PMC2783167; doi:10.1186/1471-2164-10-536)
Supplement: Additional file 4 — Abbreviations of additional file 3. Abbreviations used in the pairwise identity plots in additional file 3. [file 1471-2164-10-536-S4.DOC]

Abbreviations used in the pairwise identity plots.

**Species replicon abbreviations**

*Rhodobacter sphaeroides* 2.4.1 plasmid pB Rsphaeroides_pB

*Rhodobacter sphaeroides* 2.4.1 plasmid pD Rsphaeroides_pD

*Mesorhizobium* sp. BNC1 plasmid p2 Msp.BNC1_p2

*Rhizobium leguminosarum* bv. viciae 3841 plasmid pRL7 Rlegum_pRL7

*Rhizobium leguminosarum* bv. viciae 3841 plasmid pRL8 Rlegum_pRL8

*Rhizobium leguminosarum* bv. viciae 3841 plasmid pRL12 Rlegum_pRL12

*Rhizobium etli* CFN 42 plasmid p42f Retli_p42f

*Rhizobium etli* CFN 42 plasmid p42c Retli_p42c

*Rhizobium etli* CFN 42 plasmid p42d Retli_p42d

*Dinoroseobacter shibae* DFL 12 plasmid pDSHI04 Dshibae_pDSHI04

*Ochrobactrum anthropi* ATCC 49188 chromosome 2 Oanthropi_chromo2

*Ochrobactrum anthropi* ATCC 49188 plasmid pOANT02 Oanthropi_pOANT02

*Sinorhizobium medicae* WSM419 plasmid pSMED01 Smedicae_pSMED01

*Sinorhizobium medicae* WSM419 plasmid pSMED02 Smedicae_pSMED02

*Sinorhizobium medicae* WSM419 plasmid pSMED03 Smedicae_pSMED03

*Brucella melitensis* 16M chromosome 2 Bmelitensis16M_chromoII

*Mesorhizobium loti* MAFF303099 plasmid pMLa Mloti_pMLa
