# Supplementary figures and images for: Horizontal gene transfer and diverse functional constrains within a common replication-partitioning system in Alphaproteobacteria: the repABC operon
Source: BMC Genomics. 2009 Nov 18;10:536. doi: 10.1186/1471-2164-10-536 (PMC2783167; doi:10.1186/1471-2164-10-536)

## Slide 1
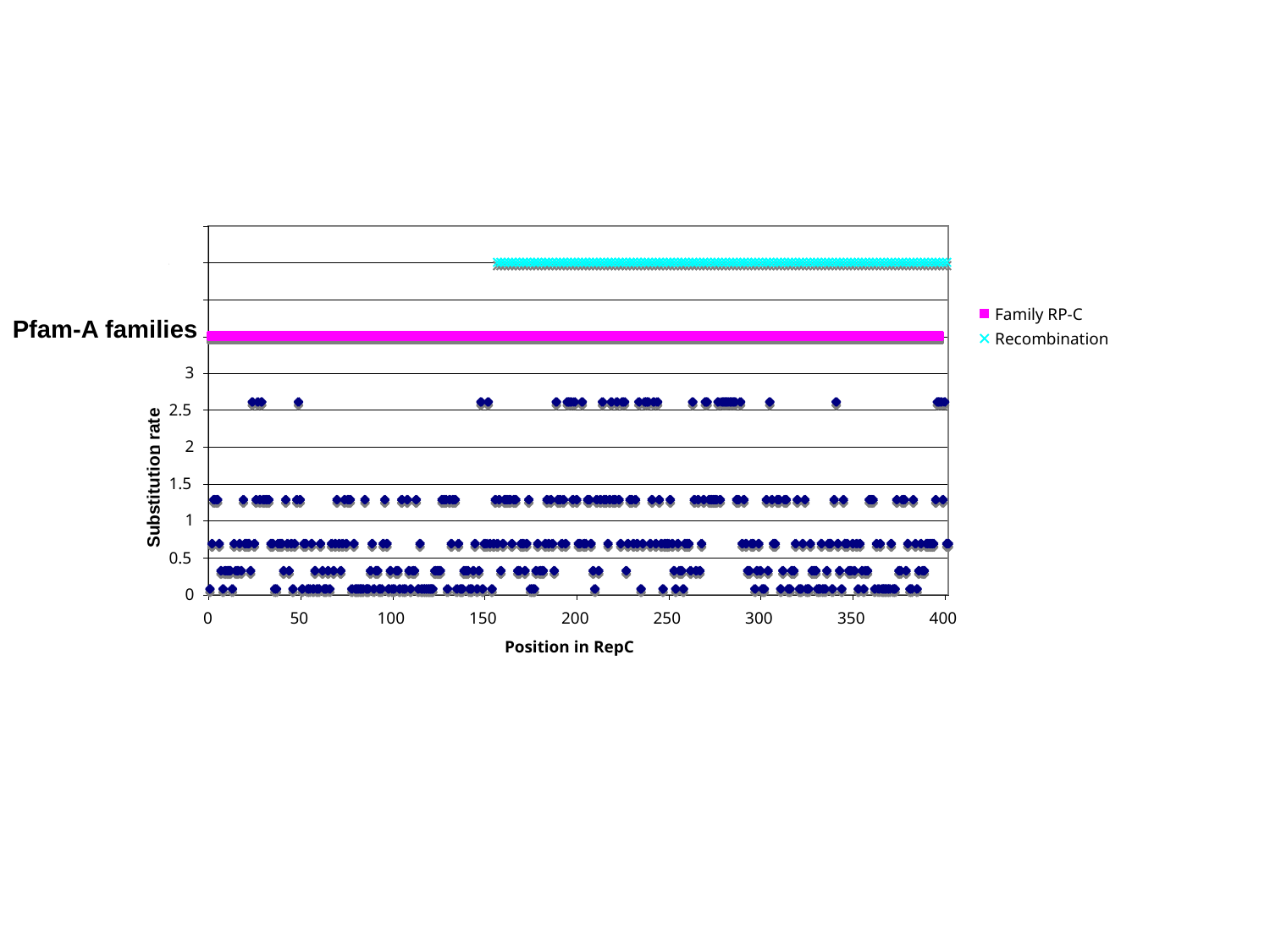

Pfam-A families
Substitution rate

Supplement: Additional file 5 — Functional restrictions within RepC. Substitution rate variation among sites in RepC. All sites were assigned to one of five gamma categories. Pfam-A domains are shown, as well as the zone affected by recombination events. [file 1471-2164-10-536-S5.PPT]
