# Supplementary material for: Horizontal gene transfer and diverse functional constrains within a common replication-partitioning system in Alphaproteobacteria: the repABC operon
Source: BMC Genomics. 2009 Nov 18;10:536. doi: 10.1186/1471-2164-10-536 (PMC2783167; doi:10.1186/1471-2164-10-536)
Supplement: Additional file 6 — Genomes used. Alphaproteobacteria genomes used to search for repABC operons. [file 1471-2164-10-536-S6.DOC]

**Genomes scanned**

*Acidiphilium cryptum* JF-5

*Agrobacterium tumefaciens* str. C58

*Anaplasma marginale* str. St. Maries

*Anaplasma phagocytophilum* HZ

*Azorhizobium caulinodans* ORS 571

*Bartonella bacilliformis* KC583

*Bartonella henselae* str. Houston-1

*Bartonella quintana* str. Toulouse

*Bartonella tribocorum* CIP 105476

*Beijerinckia indica* subsp. indica ATCC 9039

*Bradyrhizobium japonicum* USDA 110

*Bradyrhizobium* sp. BTAi1

*Bradyrhizobium* sp. ORS278

*Brucella abortus* S19

*Brucella abortus* biovar 1 str. 9-941

*Brucella canis* ATCC 23365

*Brucella melitensis* 16M

*Brucella melitensis* biovar Abortus 2308

*Brucella ovis* ATCC 25840

*Brucella suis* 1330

*Brucella suis* ATCC 23445

Candidatus *Pelagibacter* *ubique* HTCC1062

*Caulobacter crescentus* CB15

*Caulobacter* sp. K31

*Dinoroseobacter shibae* DFL 12

*Ehrlichia canis* str. Jake

*Ehrlichia chaffeensis* str. Arkansas

*Ehrlichia ruminantium* str. Gardel

*Ehrlichia ruminantium* str. Welgevonden

*Erythrobacter litoralis* HTCC2594

*Gluconacetobacter diazotrophicus* PAl 5

*Gluconobacter oxydans* 621H

*Hyphomonas neptunium* ATCC 15444

*Jannaschia* sp. CCS1

*Magnetospirillum magneticum* AMB-1

*Maricaulis maris* MCS10

*Mesorhizobium loti* MAFF303099

*Mesorhizobium* sp. BNC1

*Methylobacterium extorquens* PA1

*Methylobacterium radiotolerans* JCM 2831

*Methylobacterium* sp. 4-46

*Neorickettsia sennetsu* str. Miyayama

*Nitrobacter hamburgensis* X14

*Nitrobacter winogradskyi* Nb-255

*Novosphingobium aromaticivorans* DSM 12444

*Ochrobactrum anthropi* ATCC 49188

*Orientia tsutsugamushi* Boryong

*Orientia tsutsugamushi* str. Ikeda

*Paracoccus denitrificans* PD1222

*Parvibaculum lavamentivorans* DS-1

*Rhizobium etli* CFN 42

*Rhizobium leguminosarum* bv. viciae 3841

*Rhodobacter sphaeroides* 2.4.1

*Rhodobacter sphaeroides* ATCC 17025

*Rhodobacter sphaeroides* ATCC 17029

*Rhodopseudomonas palustris* BisA53

*Rhodopseudomonas palustris* BisB18

*Rhodopseudomonas palustris* BisB5

*Rhodopseudomonas palustris* CGA009

*Rhodopseudomonas palustris* HaA2

*Rhodospirillum rubrum* ATCC 11170

*Rickettsia akari* str. Hartford

*Rickettsia bellii* OSU 85-389

*Rickettsia bellii* RML369-C

*Rickettsia canadensis* str. McKiel

*Rickettsia conorii* str. Malish 7

*Rickettsia felis* URRWXCal2

*Rickettsia massiliae* MTU5

*Rickettsia prowazekii* str. Madrid E

*Rickettsia rickettsii* str. 'Sheila Smith'

*Rickettsia rickettsii* str. Iowa

*Rickettsia typhi* str. Wilmington

*Roseobacter denitrificans* OCh 114

*Silicibacter* *pomeroyi* DSS-3

*Silicibacter* sp. TM1040

*Sinorhizobium medicae* WSM419

*Sinorhizobium meliloti* 1021

*Sphingomonas wittichii* RW1

*Sphingopyxis alaskensis* RB2256

Wolbachia endosymbiont of Drosophila melanogaster

*Wolbachia endosymbiont* strain TRS of Brugia malayi

*Xanthobacter autotrophicus* Py2

*Zymomonas mobilis* subsp. mobilis ZM4
